# Acquired epidermodysplasia verruciformis syndrome in HIV-infected patients: a systematic review

**DOI:** 10.1007/s00403-024-03016-x

**Published:** 2024-07-13

**Authors:** Daniel Cuestas, Alexa Gómez, Constanza Neri Morales, Adriana Motta, Mariam Rolon, Samanda Suarez, Rosa Polo

**Affiliations:** 1 Universidad de La Sabana, Campus Puente Común Km. 7, Autopista Norte de Bogotá, Chía, Colombia; 2 Estudioderma - Dermatologic Investigation Center - Medical Research Area, Bogotá, Colombia; 3 Dermatology Department, Universidad el Bosque, Bogotá, Colombia; 4 Universidad del Sinú, Cartagena, Colombia; 5 Universidad de los Andes, Bogotá, Colombia; 6 Universidad Rey Juan Carlos, Madrid, Spain

**Keywords:** HIV, Epidermodysplasia verruciformis, Skin cancer, HPV

## Abstract

Congenital epidermodysplasia verruciformis (CEV) is a Genodermatosis linked to different inheritance patterns and mutations of the EVER1/TMC6 and EVER2/TMC8 genes. There is an acquired form (AEV) associated with immunodeficiency states, including human immunodeficiency virus (HIV) infection; however, the literature about AEV is limited and imprecise, so a systematic review was performed. A search of the main databases from 1975 to 2021 identified 126 studies, of which 80 met the inclusion criteria. The diagnosis of AEV is complex due to atypical manifestations and locations, it requires a mean follow-up of 7 years, and the lesions do not change with ART therapy, CD4 count, or viral load. Histopathological findings are variable depending on the location of the lesions. HPV 5 remains the serotype most frequently associated with AEV and CEV, although HPV 20 is more frequent than HPV 8 in AEV. Most treatments have low efficacy, the most described are glycolic acid 15%, 5-fluorouracil 5%, imiquimod 5%, and topical retinoids all of them in monotherapy or combined with cryotherapy. Other alternatives include topical cidofovir and systemic retinoids with variable results. The oncologic prognosis is still inconclusive; however, the development of squamous cell carcinoma and melanoma are frankly lower concerning CEV. This review opens new opportunities for future research. Additionally, we provide clear and useful key points for the practice of dermatologists and all professionals treating HIV patients around the world.

**Supplementary Information:**

The online version contains supplementary material available at 10.1007/s00403-024-03016-x.

## Introduction

Congenital epidermodysplasia verruciformis (CEV) is a genodermatosis described by Lewandowsky and Lutz in 1922 [1–3]. Several patterns of inheritance have been described in CEV, linked to mutations in the EVER1/TMC6 (GenBank 11,322) and EVER2/TMC8 (GenBank 147,138) genes located on chromosome 17q25. 3, which increase susceptibility to infection by specific subtypes of human papillomavirus (HPV) predominantly of the beta group, including more than 20 types (3, 5, 8, 9, 10, 10, 12, 14, 15, 17, 19–25, 28, 29, 36, 46, 47, 49 and 50, among others) that do not normally cause infection in healthy individuals [1–3].

The term **acquired EV** (AEV) was introduced by Rogers et al. (2009) when it was shown that not only genetic alterations explain the susceptibility for the development of epidermodysplasia verruciformis, but also acquired immunodeficiency states such as solid organ transplantation, human immunodeficiency virus (HIV), severe acquired combined immunodeficiency (SCID), lepromatous leprosy, Hodgkin’s lymphoma, systemic lupus erythematosus (SLE), WILD syndrome (warts, immunodeficiency, lymphoedema, and anogenital dysplasia), IgM deficiency, T-cell leukemia, graft-versus-host disease and some biologic therapy patients [1–3].

The literature on HIV-associated AEV is limited to case reports, case series, not systematic-narrative reviews, and letters to the editor. Information regarding clinical features, prognosis, behavior with or without antiretroviral therapy, impact of viral load, CD4 count, differentiating histological findings with epidermodysplasia verruciformis, and whether an underlying molecular context exists in these patients is not fully established. In this systematic review, we provide a clear and precise overview with 10 practical points in the care of HIV patients.

## Methods

A systematic review was carried out in principal databases (Clinical Key, Elsevier Science, Embase, Europe PMC, Google Scholar, Medline, Ovid, PubMed, Scielo, Scopus, Web of Science) from 1975 to January 2021, without any language restrictions, including case reports (≤ 5 cases in the same publication), case series (≥ 6 cases in the same publication), observational studies, clinical trials, systematic and nonsystematic reviews, meta-analyses, and gray literature complementary manual search.

**Fig. 1:**
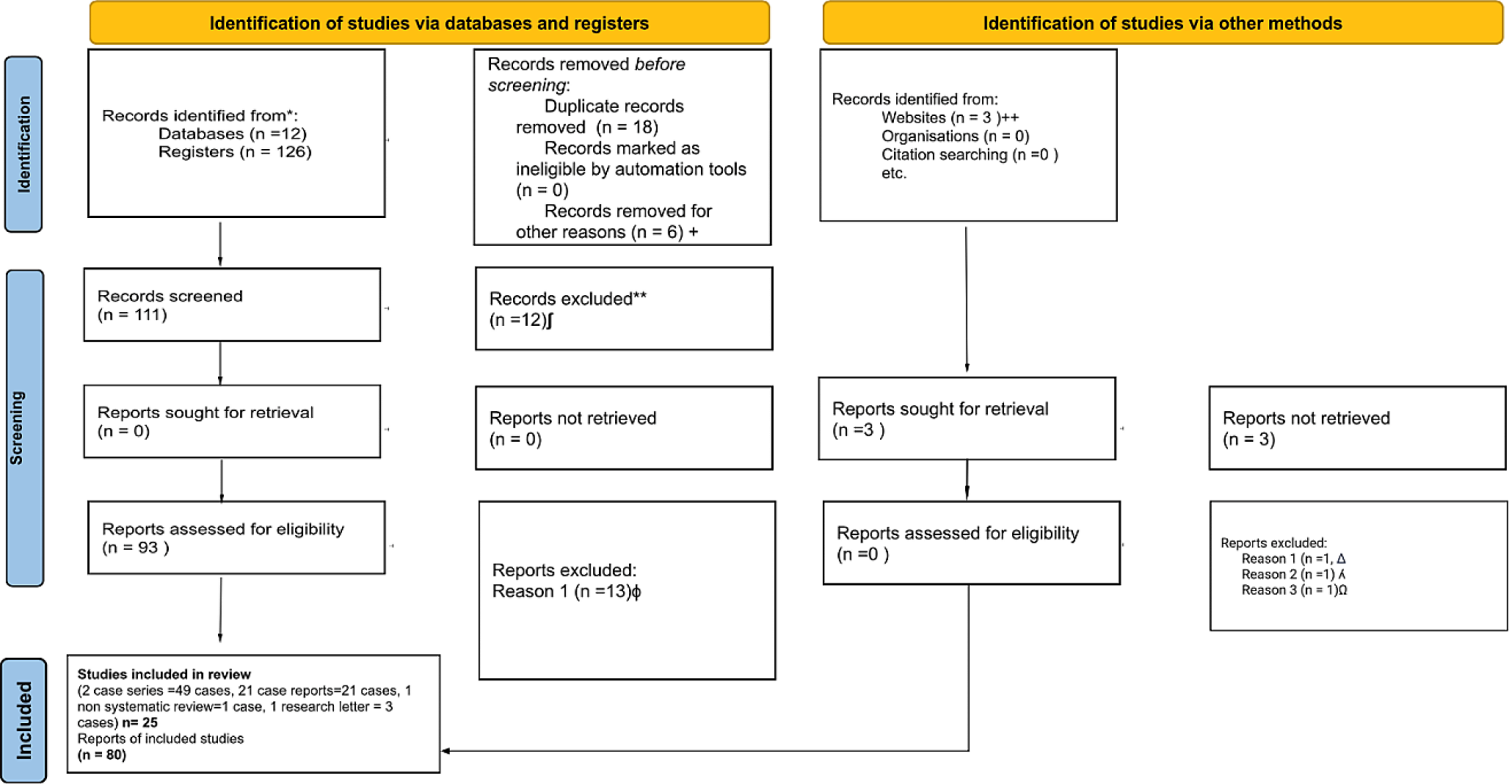
PRISMA 2020 flow diagram for new systematic reviews. The search time, filtering, statistical application *p* < 0.05, and hypothesis verification by Shapiro Wilk test was 1 year. (ʃ) First stage of exclusion, the title or abstract does not match the case description and does not meet the inclusion criteria. (++) Grey literature (+) Title or abstract does not meet eligibility criteria (Δ) Old Documents or Unavailable Sources). (ʎ) Lack of Response (Ω) Different Format. (ɸ) Cases that included AEV-related keywords but did not meet the inclusion criteria. (*) Records identified from each database or register searched (rather than the total number across all databases/registers). (**) If automation tools were used, indicate how many records were excluded by a human and how many were excluded by automation tools. *According to* MJ, McKenzie JE, Bossuyt PM, Boutron I, Hoffmann TC, Mulrow CD, et al. The PRISMA 2020 statement: an updated guideline for reporting systematic reviews. BMJ2021;372:n71. DOI: 10.1136/bmj.n71

The search was carried out using four six clusters; the first one included (HIV): OR (acquired immunodeficiency syndrome), OR (AIDS). The second one included (epidermodisplasia verruciforme): OR (**Epidermodysplasia verruciformis**), OR (**Lewandowsky–Lutz dysplasia**), OR (**treeman syndrome**), OR (**epidermodisplasia verruciforme de Lutz-Lewandowsky**). The third cluster included (EVER1/TMC6, EVER2/TMC8). The fourth cluster included (HPV): OR (human papillomavirus), OR (VPH), OR (viral warts), and OR (clinical findings). The fifth cluster included (histology): OR (pathological findings). The sixth cluster included (CD4 count): AND (viral load), OR/AND (HAART) OR/AND (ART), OR (**antiretroviral therapy).** Each cluster was combined in the different databases described. Abstracts and full manuscripts were reviewed by applying PRISMA guidelines screening criteria for eligibility (PRISMA Flow Diagram—Fig. 1). The complete search took 2 years between 2019 and 2021.

The quality of the publications was analyzed with the Care Check (2013) modified Delphi Quality Appraisal Checklist (2018), AMSTAR2 (2017) and STROBE Checklist (2007). These same recommendations were suggested by The EQUATOR (Enhancing the Quality and Transparency of Health Research) Network.

Exclusion criteria corresponded to cases of CEV, those related to non-HIV-associated infectious, pharmacological, autoimmune, and neoplastic causes. Inclusion criteria corresponded to all patients diagnosed with exclusively HIV-associated AEV regardless of age.

## Results

We documented 80 cumulative cases between 2 case series (49 cumulative cases), 21 case reports (27 cumulative cases), 1 non-systematic review (1 cumulative case), and 1 letter to the editor (3 cumulative cases) (Fig. 1) [1–27].

### Origin of publications and origin of individuals with AEV

Most cases correspond to publications from the United States of America (82%), followed by Spain (5%) and the United Kingdom (3.75%) (Table 1).

**Table 1:** Total number of publications by country and their respective percentage

Countries of Publication	USA	Spain	United Kingdom	Canada	Switzerland	Berlin	Brazil	Japan
Total	66	4	3	2	2	1	1	1
Percentage	82.5	5.00	3.75	2.50	2.50	1.25	1.25	1.25

However, it is notable that most patients described in the publications come from the Republic of Botswana, a landlocked country in Southern Africa (47%), followed by the USA (15%), France, and Spain (6.25%), see supplementary files 1 and 2.

### Age and sex in patients with AEV

70% of the individuals were male and 30% female. The 75% percentile age was 40 years with a mean age of 25 (±) 15 years. 28.8% of the patients were in the pediatric age range below 18 years, with 2 patients aged 7 years and 4 patients aged 16 years being at the extremes of this range [1–27].

### Clinical manifestations

The clinical features of CEV include hypopigmented macules, red-brown pityriasis versicolor, and flat wart-like papules, located mainly on the trunk, neck, arms, and face during childhood. Over time, in half of the individuals, these lesions may acquire dysplastic changes, especially in sun-exposed areas [1–27].

In contrast, in our review, we identified that AEV presents with flat warts (32.5%), followed by macules, papules, or pink or brown plaques, hypo- or hyperpigmented between 1 and 4 mm (16.25%), located predominantly on the face, upper limbs and upper trunk, and to a lesser extent on the lower limbs (8.75%) and anogenital area (3.75%). However, it is important to mention that 47.5% of the published cases had no clinical descriptions. Pityriasis versicolor-type eruptions were found in a lower proportion (11.25%), with the upper trunk and upper extremities followed by the lower limbs. Other lesions such as lichenoid papules (5%) and seborrheic keratosis-like keratotic plaques (3.75%) were less frequently described [1–27]. Finally, 97.5% of the publications did not specify associated symptomatology, only 1 case described being truly asymptomatic, and 1 case associated it with itching.

The clinician should keep in mind that up to 12.5% of patients with HIV before AEV diagnosis may have flat warts and that after ART initiation the changes were minimal, making them less palpable [1–27].

### Comorbidities and infections

Overall, a low percentage of comorbidities was found; the most described comorbidities included diffuse large B-cell lymphoma (3.75%), congenital hemophilia B (1.25%), Hodgkin’s disease (1.25%), multicentric Castleman’s disease (1.25%) and psoriasis (1.25%). Non-infectious comorbidities were not specified in 73% of the cases described [3, 5, 8, 11].

Regarding co-infections, the most frequently found co-infection was hepatitis C virus (3.75%), followed by hepatitis B virus (2.5%), Pneumocystis jirovecii (1.25%), syphilis (1.25%), and mycobacterium tuberculosis infection (1.25%) [3, 5, 7, 8, 15].

### Skin biopsy and histopathological aspects

Skin biopsy is usually taken from flat viral warts, most frequently located in areas of easy access and scarring, such as the back of the hands (31.25%) and the face (8.75%), and to a lesser extent the trunk (3.75%) and lower extremities (1.25%). However, a significant percentage of publications did not specify the site of biopsy and the type of lesions biopsied (42.5%) [1–27].

Histopathological findings were described in 27.5% of the cases, while in 8.75% of the cases, no histopathological findings were described despite sampling. In 62.5% of cases, no biopsy was performed. Findings include uniformly thickened epidermis with parakeratosis, acanthosis, and hypergranulosis interposed between segments of normal epidermis. The epidermis may exhibit large keratinocytes with pale stained, finely granular cytoplasm and voluminous vacuolated nuclei with the presence of mitotic figures. These cells occupy most of the epidermis in a banded arrangement. Usually, no changes are found at the dermo-epidermal junction or in the dermis. Occasionally samples may show cytological atypia and prominent vascular plexuses. Electron microscopic examination reveals numerous viral particles in the nuclei of the upper keratinocytes; particles up to 40 nm in size with a geometric, crystalline, and sometimes hexagonal pattern. In our study, we found only 1 case report with electron microscopic examination [1–27].

Immunohistochemistry studies were not performed in 85% of the cases found, and in 11.25% of the cases, there were no descriptions of the performance of this study [4, 5, 21]. The presence of dysplasia was detected in a very low percentage (3.75%). In 62.5% of the cases, it was not possible to assess the presence of dysplasia or malignant degeneration, as they correspond to cases in which no biopsy was performed [4–6].

### HPV typing

HPV typing was performed in 73.5% of cases. HPV 5 was most frequently detected (26.5%) followed by HPV 20 (23.75%), HPV 8 (13.75%), and HPV 25 (12.5%). Other serotypes found in lower percentages were 3, 6, 11, 14–16, 17, 19, 22–26, 36–38, 43, 47, 52 and 65. Additionally, other distinct, yet undefined HPV types obtained from NCBI-GenBank, including DL287a, DL416a, FA75a/KI88-03a, GRT04a, HPV 96, HPVX34a, DL231a, and RTRX9a, were also reported [1–27].

### Molecular study of EVER1 and EVER 2 in AEV patients

Only 5 individuals were found to have molecular studies for associated mutations in EVER1 and EVER2 in the context of AEV in HIV patients. Of these, 2 patients were found to have HLA II haplotypes DQB1*0301, DRB1*11, and DQA*0501. We reported one case with polymorphism in EVER2 consisting of c917A-T, and one case with the variants rs2748427 in TMC6 and a homozygous isoleucine at position 125 of TMC8 (rs7208422) [17, 22, 28].

### Local and systemic treatments

Local and systemic treatments are described, sometimes they can be combined. Local ones include glycolic acid 15% (47%), cryotherapy + retinoic acid + topical imiquimod + urea + topical 5-fluorouracil (15%), topical retinoic acid monotherapy (5%), topical imiquimod 5% monotherapy (3.75%), cidofovir (2.5%) and topical 5-fluorouracil 5% monotherapy (1.25%). Other less frequent combinations include cryotherapy + topical imiquimod and cryotherapy + topical imiquimod + cidofovir (1.25%) [1–27].

Clinical response with monotherapy was variable, resolving lesions in 13.1% with 15% glycolic acid (38 individuals), 50% with imiquimod (3 individuals), 75% with topical cidofovir (3 individuals), and 25% with topical retinoids (4 individuals) [1–27].

Regarding systemic treatments, 2 cases are reported by different authors [22, 24]. With experience and partial response with acitretin 20–50 mg and a case of management with subcutaneous interferon alfa-2a [8]. However, none of the local or systemic treatments have shown clearly effective results, since although many of these options induce improvement, in the follow-up there has been evidence of recurrence or rapid growth of new lesions after the end of treatment [1–27].

### Antiretroviral therapy

Most patients were on lamivudine (40%), zidovudine (33.75%), and tenofovir disoproxil fumarate (18.75%). Other forms of ART included efavirenz, atazanavir, raltegravir, zalcitabine, lopinavir, ritonavir, didanosine, nevirapine, stavudine, emtricitabine, and indinavir. However, none of the reports accurately describe the precise time or timing of ART initiation or whether any changed the clinical course of AEV. In the publication by Jacobelli et al. [3] it is mentioned that the initiation of ART was ineffective during skin lesions [1–28].

### AEV behavior in HIV

The 75th and 100th percentile of time in years for individuals with HIV was 22 to 24 years with an average of 12 ± 9.7 years. The time between HIV diagnosis and AEV onset at the 75th percentile was after 5 years and the 100th percentile was after 16 years. However, the mean of these data is distant due to the isolated reports of cases in which a late diagnosis of AEV was made. The time course of AEV to final diagnosis was 5.7 ± 4.7 years (18 individuals). The mean CD4 count before the onset of AEV was 348 (17 individuals), while the count after the diagnosis of AEV was 449 (68 individuals); concerning the viral load before the onset of AEV was 9075 (12 individuals) to the time of presence of AEV with 31,418 (65 individuals) in this systematic review (Table 2). This difference considers the lack of CD4 count and viral load descriptions in the publications.

**Table 2:** Percentiles and averages with absolute values of time to HIV diagnosis (HIV.TIME), the time between HIV and AEV (HIV AEV.time), AEV.time (Diagnostic timing), Number of skin biopsies linked with AEV diagnosis (NumbnSB), CD4 Cell count during diagnosed HIV (CD4cDXHIV), CD4 cell count during the appearance of AEV (CD4AEV), HIV RNA copies during diagnosed HIV (RNAcDXHIV), HIV RNA copies during appearance of AEV lesions (RNAcAEV), standard deviation (sd), SWP = Shapiro Wilk test

Variable	p0	p0.25	p0.5	p0.75	p1	mean	sd	NAs	*n*	SWp
Age	7	12,47	15,5	40,25	58	25,047	15,1239	0	80	0,00000004894396857
HIV.Time	0	0	13	22	24	12,2759	9,783	51	29	0,0002354063213
HIV AEV.time	0	0,25	5	16	27	8,0076	8,303	47	33	0,0002906958415
AEV.time	0,25	2	5,5	7,75	20	5,7917	4,7791	62	18	0,01671536571
NumbnSB	0	1	1	1	8	1,1831	0,9458	9	71	0
CD4cDXHIV	6	78	304	536	1040	348,5294	286,3187	63	17	0,1394493962
CD4AEV	2	265,75	544,28	544,28	1115	449,3476	208,5601	12	68	0,0000002710544959
RNAcDXHIV	50	50	50	127,1945	108,000	9075,2438	31153,2721	68	12	0,000001228577865
RNAcAEV	40	4950	42,009	42,009	82,000	31418,3255	21256,748	15	65	0,000000006033859226

Regarding the development of lesions in AEV cases, there appears to be no relationship with CD4 count or viral load, as it was observed that lesions can develop within a wide range of CD4 counts (between 2 and 767) and viral loads (< 50 to 623,000) [3].

### Oncologic prognosis

Regarding the prognosis of patients with CEV, some HPV types have been found to have oncogenic effects associated with a 30–60% risk of developing squamous cell carcinoma (SCC). This malignant transformation is usually slow, and neoplasms first appear in photo-exposed areas around the fourth decade of life (20–30 years after disease onset) [1–27]. The oncogenic nature of HPV and its synergistic role with ultraviolet radiation (UVR) in inducing carcinogenesis remains unclear. On the contrary, our review shows that the malignant potential in cases of AEV has not yet been characterized due to the lack of long-term follow-up of these patients [1–27]. However, our results seem to indicate that these patients present a lower oncologic risk.

## Discussion and highlights

### Socio-demographic connotation

In this systematic review, there does not appear to be any significant relationship between AEV and sex; however, the absence of data in the publications does not allow a definitive conclusion. Most of the cases described correspond to adult patients, although reports were also found in the pediatric population without associated genetic factors. Even individuals in whom haplotypes and polymorphisms related to CEV were detected, did not manifest AEV until HIV acquisition.

These findings suggest that the clinician should perform dermatologic examination of patients with HIV 5–15 years after diagnosis [1–28]. On the other hand, it is notable that most of the patients described in the publications come from the Republic of Botswana, a landlocked country in Southern Africa, followed by the USA, France, and Spain, which raises new questions regarding a genetic basis or trigger versus a lack of public health guidelines for the epidemiologic control of HIV.

**Fig. 2:**
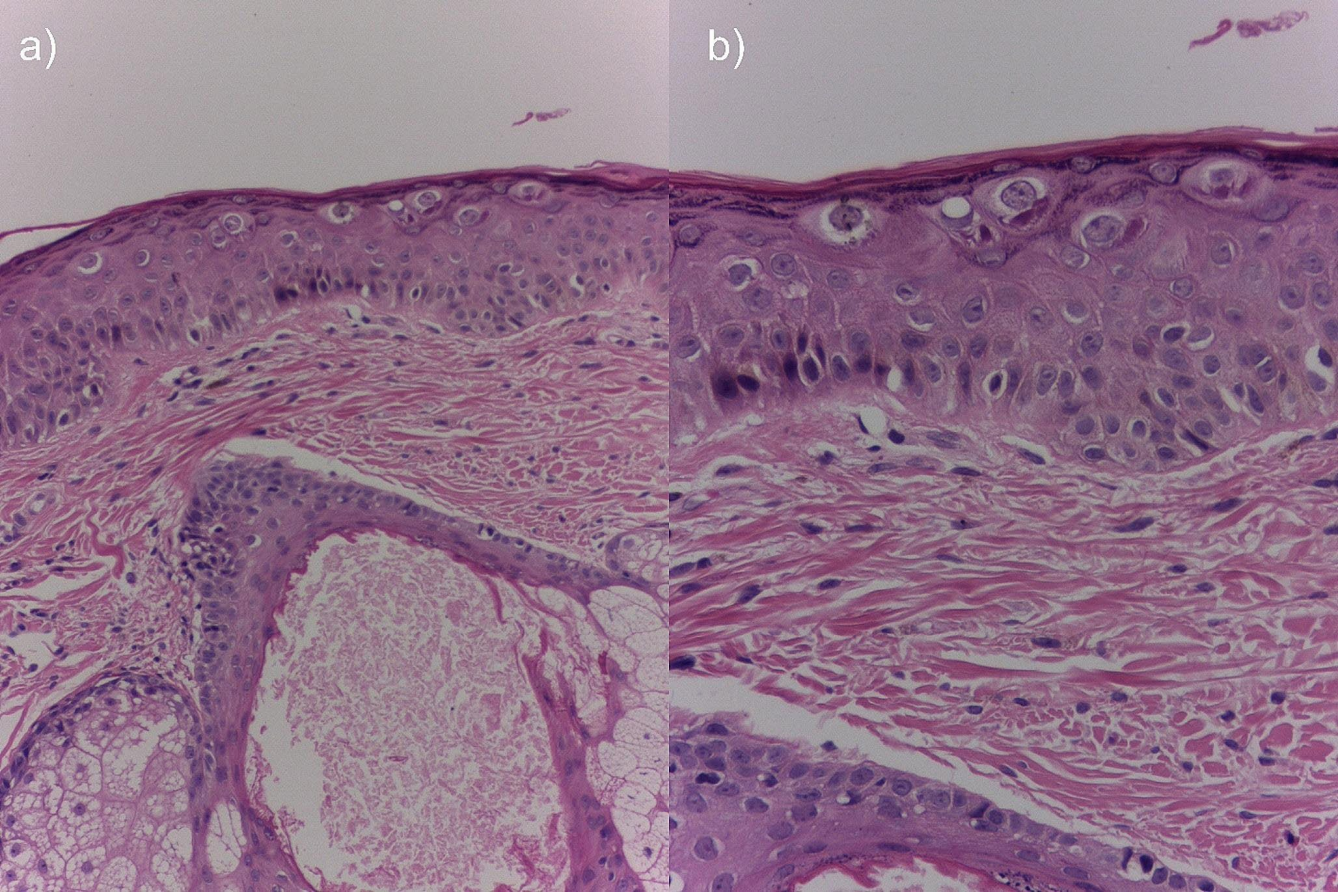
Histopathological findings in acquired epidermodysplasia verruciformis. H&E 80x (**a**) and 60x (**b**). Courtesy of Dr. Mariam Rolón

### Highlights

Given the clinical, biomolecular, histological, and therapeutic complexity, we invite the physician to consider these 12 key points for the medical practice [1–28].

I) In patients with HIV, viral warts that develop after ART initiation are more difficult to detect in a non-dermatological consultation as they are less palpable and visible in contrast to the hypochromic papules or plaques that develop before ART; therefore, it is the dermatologist who must assess these patients.
II) The clinician is encouraged to look for viral warts in areas not necessarily photo-exposed as this review excludes the classical theory of the role of UV in HPV-β-induced lesions. However, this data should be handled with caution, as most biopsies were taken from photo-exposed areas.
III) It is suggested to look for pityriasis versicolor-like in places distant from sun exposure and in locations where even classical pityriasis versicolor is unusual, such as the lower limbs, buttocks, and anogenital region.
IV) No statistically significant findings were found to affect the course or occurrence of AEV before neoplastic events and/or coinfections, which concludes that these events do not have a directly associative and inclusive relationship.
V) The increased survival of patients with AEV in the context of HIV reflects in the long term the possible appearance of anogenital warts and squamous cell carcinoma however the rate of dysplasia in these patients is low (3.75%) in contrast to CEV which reaches up to 50% of cases, possibly related to the earlier onset and genetic load.
VI) This study identified the low existence of dysplasia and/or malignancy, which suggests that ART therapy may have some positive impact on AEV, in contrast to CEV, which usually presents non-melanoma skin cancer (NMSC) involvement in up to 50% of cases, probably related to the earlier onset of the lesions.
VII) Histopathological confirmation and HPV typing are suggested in all patients suspected of AEV. This review reveals that in contrast to CEV, HPV 20 (23.75%) is more frequent than HPV 8 (13.75%) and implies less oncogenic capacity, however, HPV 5 continues to be the most frequent serotype in both forms of epidermodysplasia. In addition, HPV 25 (12.5%) and other serotypes documented in NCBI-GenBank should be considered in AEV, which are soon to be published. These findings will allow oncologic follow-up strategies. Additionally, we found that the presence of HLA II haplotypes may constitute a possible risk factor for HPV-β infection.
VIII) It should be noted that all mutations in patients with CEV reported to date are frameshift, nonsense, or splice-site mutations. In addition, nonsense mutations in the development of CEV remain unclear, but it appears that they may trigger an attenuated defense mechanism against certain HPV types through altered TMC functionality, thus allowing viral replication in the context of immunodeficiency. However, this conclusion remains to be defined and future research is required.
IX) Skin biopsy is usually taken from flat viral warts, most often located in areas of easy access and scarring, and there are no statistically significant differences in histology in those taken from non-photo-exposed areas.
X) This study found no statistically significant findings related to the biological course of these patients and variability in CD4 count and/or viral load. Likewise, we analyzed the behavior of the disease according to the ART therapy of each case and/or cases, finding clinical variability.
XI) The dermatologist or dermatopathologist should consider the following possible findings for the diagnosis of AEV: uniformly thickened epidermis with parakeratosis, acanthosis, or hypergranulosis interposed between segments of normal epidermis. The epidermis may exhibit large keratinocytes with pale stained, finely granular cytoplasm and voluminous vacuolated nuclei with the presence of mitotic figures. These cells occupy most of the epidermis in a banded arrangement. Usually, no changes are found at the dermo-epidermal junction or in the dermis. Occasionally the specimens may show cytologic atypia and prominent vascular plexuses. (Fig. 2).
XII) Regarding treatment, no therapy has demonstrated efficacy, and data is limited to case reports and small studies. Some topical or local treatments include imiquimod, glycolic acid, cidofovir, and/or retinoids as first-line management. Second-line treatments include systemic retinoids or interferon. It is important to note that although the lesions resolve, they may recur if therapy is discontinued.

## Study limitations and bias reduction

The limitations of the research correspond to variations in the methodological quality of each study or publication, which may include a lack of detail in the clinical histories, dermatologic findings, absence of histologic, genetic, HPV typing, CD4 count, and/or viral load, and response to treatment. Therefore, the reader should exercise caution with the handling of these data.

To reduce bias, the following considerations were considered: (i) a review of each publication and application of the reporting guidelines and (ii) a critical Evaluation of the quality of the reports. In this regard, it was determined that of all reports 9.8% were of high quality, 58.2% of medium quality, 21.3% of low quality, 0.8% of poor quality, and 9.8% without full access for evaluation; (iii) public access to positive and negative data found during the study to reduce the risk of “publication bias”; and (iv) inclusion of gray literature, which is not available in traditional databases such as PubMed/MEDLINE, EMBASE and Web of Science.

The variability and heterogeneity of the data can be explained by the lack of reporting guidelines in each journal. Publications before 2003, 2007, and 2012 (EQUATOR Networks) did not have a standardized reporting or evaluation model.

## Conclusions

This systematic review covers all publications on HIV-associated acquired epidermodysplasia verruciformis, providing a clearer picture of sociodemographic, clinical, genetic, histopathologic, phenotypic, and therapeutic aspects, which opens windows for future research related to genetic and phenotypic identification of patients at risk for AEV, as well as on therapeutic advances in AEV. Additionally, we provide useful clinical data for dermatologic practice and for professionals who care for patients with HIV daily.

## Electronic supplementary material

Below is the link to the electronic supplementary material.

Supplementary Material 1

Supplementary Material 2

## Data Availability

Data is provided within the supplementary information files.
